# Effect of Pristine Graphene on Methylammonium Lead Iodide Films and Implications on Solar Cell Performance

**DOI:** 10.1021/acsaem.1c02738

**Published:** 2021-11-17

**Authors:** C. Redondo-Obispo, P. Serafini, E. Climent-Pascual, T.S. Ripolles, I. Mora-Seró, A. de Andrés, C. Coya

**Affiliations:** † Instituto de Ciencia de Materiales de Madrid, Consejo Superior de Investigaciones Científicas, C/Sor Juana Inés de la Cruz 3, 28049 Madrid, Spain; ‡ Institute of Advanced Materials (INAM), Universitat Jaume I, 12071 Castelló, Spain; § Escuela Técnica Superior de Ingenieros Industriales, Universidad Politécnica de Madrid, C/José Gutiérrez Abascal 2, 28006 Madrid, Spain; ∥ Escuela Técnica Superior de Ingeniería de Telecomunicación, Universidad Rey Juan Carlos, C/Tulipán s/n, 28933 Madrid, Spain

**Keywords:** hybrid perovskites, graphene, XRD, photostability, ambient stability, impedance spectroscopy, porosity

## Abstract

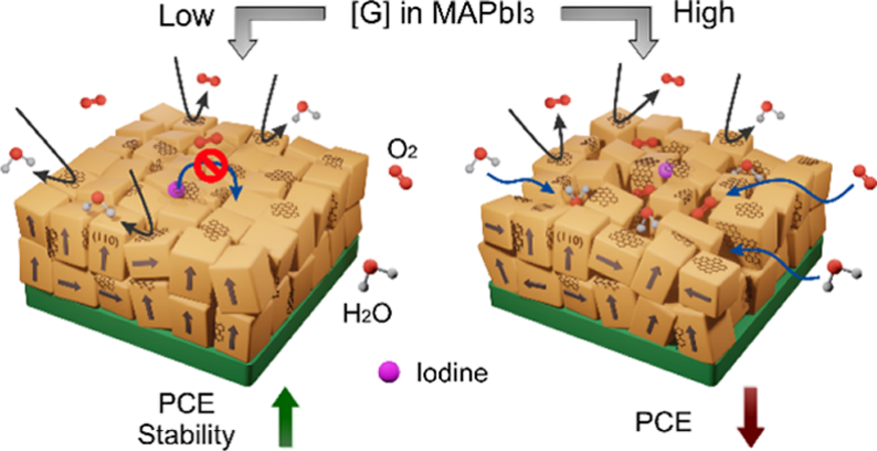

The relatively low stability of solar cells based on hybrid halide perovskites is the main issue to be solved for the implementation in real life of these extraordinary materials. Degradation is accelerated by temperature, moisture, oxygen, and light and mediated by halide easy hopping. The approach here is to incorporate pristine graphene, which is hydrophobic and impermeable to gases and likely limits ionic diffusion while maintaining adequate electronic conductivity. Low concentrations of few-layer graphene platelets (up to 24 × 10^–3^ wt %) were incorporated to MAPbI_3_ films for a detailed structural, optical, and transport study whose results are then used to fabricate solar cells with graphene-doped active layers. The lowest graphene content delays the degradation of films with time and light irradiation and leads to enhanced photovoltaic performance and stability of the solar cells, with relative improvement over devices without graphene of 15% in the power conversion efficiency, PCE. A higher graphene content further stabilizes the perovskite films but is detrimental for in-operation devices. A trade-off between the possible sealing effect of the perovskite grains by graphene, that limits ionic diffusion, and the reduction of the crystalline domain size that reduces electronic transport, and, especially, the detected increase of film porosity, that facilitates the access to atmospheric gases, is proposed to be at the origin of the observed trends. This work demonstrated how the synergy between these materials can help to develop cost-effective routes to overcome the stability barrier of metal halide perovskites, introducing active layer design strategies that allow commercialization to take off.

## 1 INTRODUCTION

The goal of lowering solar electricity costs by improving the efficiency, reliability, and durability of emerging metal halide perovskite (MHP) solar cells (PSCs) is actually an intense area of research. Three-dimensional (3D) MHPs are described by the general formula ABX_3_, where A, B, and X are a monovalent cation (MA: CH_3_NH_3_^+^, FA: NH_2_CH = NH_2_^+^, and Cs^+^), a divalent metal cation (mainly Pb^2+^), and a halide anion (Br^–^, Cl^–^, or I^–^), respectively.^1^ PSCs have already demonstrated outstanding power conversion efficiencies (PCEs), reaching a certified 25.5% efficiency^2^ of photovoltaic devices for 3D Pb-based perovskites in record time. In just 1 decade, PSCs have surpassed CIGS and CdTe-based solar cells but using low-temperature and cost-effective methods as solution processing, revolutionizing existing technologies with simple layered-structure devices.^1^ MHPs exhibit high absorption coefficients^3^ and sharp absorption edges,^4^ long charge carrier diffusion length,^5^ low exciton binding energy,^6^ and tunable band gap in the ideal range for silicon-based tandem devices (1.5–1.8 eV).^7^ However, despite this potential, significant efforts are still needed to overcome various technological challenges, such as very high instability under environmental conditions. Moisture, air, UV light, thermal stress (heat), light soaking, and electric fields, among other agents, degrade the perovskite solar cells,^8,9^ especially outdoors,^10^ and prevent market requirements from being achieved. MHP rapidly evolves to PbI_2_ or PbO_*x*_ in the presence of moisture or light irradiation.^11−13^ Even in an inert atmosphere, the most representative perovskite absorber material, MAPbI_3_, appears to be thermally unstable as revealed by the observed transformation into PbI_2_.^14^ Stability is therefore one of the main barriers preventing the widespread application of PSCs.^15,16^

Most of the advances in the performance of PSCs have resulted from improvements in device architecture and material composition. The excellent properties that MHPs provide on the one hand, and those of graphene (G) on the other, can envision great enhancement of device properties. The hydrophobic character of G^17^ could enhance stability and passivation of traps at the crystalline domain interfaces, or better energy level alignment^18^ could improve charge injection/extraction that could lead to more efficient devices. In fact, the incorporation of graphene-related materials (GRMs) in PSC devices has been demonstrated to be a promising route toward applications and improvements in performance and stability and, very importantly, for their commercial launch.^19−22^ It has been observed that covering a two-dimensional (2D) perovskite with a graphene monolayer maintained reasonable optical properties after exposure to high temperature and humidity.^23^ Note that pristine graphene (sp^2^ C) and functionalized graphene [such as graphene oxide (GO), reduced GO (rGO), G quantum dots (GQDs), and other derivatives further functionalized] have different properties and behaviors related to the presence of oxygen (or other) functional groups.

The literature describes how GO or rGO is used to dope or substitute the hole transport layer (HTL) and the use of G, GQDs, Li-GO, and rGO in the electron transport layer (ETL), with a positive effect on both charge extraction rates and stability of PSCs. However, the combination/incorporation of GRM into the active layer is less frequent and extremely scarce are the works that incorporate pristine graphene. Morphology, grain size, and thickness are critical factors in the charge dynamics and the final performance of the device that can be modified by the use of this GRM. Among them, carbon nanotubes have been used as additives in the perovskite active layer as electron donors or acceptors to reduce charge recombination, acting as a nucleation template.^24^ Nitrogen-doped rGO, N-rGO, has been added to obtain bigger crystal domains to passivate the surface and to delay the recombination of charges.^25,26^ Eventually amine-functionalized-G^27^ and GQDs^28,29^ have been added to improve charge-extraction efficiency and facilitate charge transport. Chen et al.^30^ simultaneously incorporated N, S codoped GQDs in the ETL-perovskite interface and into the perovskite active layer of the PSC acting as a nucleating template, easing charge extraction and defect passivation, obtaining efficiency and stability improvements of the device. Also, electrospun graphene nanofibers^31^ added into the perovskite improved nucleation and crystallization at the nanofiber interface, favoring charge transport and resulting in improved photovoltaic performance and stability of the final device. Recently, Lou et al.^32^ used a graphene/halogenated-polymer composite dispersion as an antisolvent in the perovskite synthesis, observing that halogen elements (Cl/F) in the polymer can p-dope the graphene and endow it with higher hole-transport selectivity. However, it should be noted that, to our knowledge, the effect of only pristine graphene platelets on the active perovskite layer has not been investigated and until now they have been hardly used in the charge-transport layers. Wang et al.^33^ and Agresti et al.^34^ tested nanocomposites of G and TiO_2_ nanoparticles and doped the mesoporous TiO_2_ layer with graphene flakes, respectively, and reported charge-carrier injection and collection improvements and cell stability. Recently, we have demonstrated a route for doping poly(3,4-ethylenedioxythiophene)–poly(styrenesulfonate) (PEDOT:PSS) HTL with pristine G-nanoplatelets, obtaining significant improvement on conductivity without the loss of transmittance at concentrations well below percolation.^35^ The integration of this G-doped HTL into inverted MAPbI_3_ solar cells resulted in more efficient devices due to an enhancement of charge extraction and a reduction of charge accumulation at the graphene–PEDOT:PSS interface as well as stability improvement under environmental conditions.

Here, we act on the hybrid lead iodide perovskite active layer, searching to exploit the synergy between these different materials. Specifically, the most commonly used hybrid halide perovskite, MAPbI_3_, is combined with pristine graphene platelets into the active layers of the solar cells. For the appropriate amount of graphene (only 0.6 × 10^–3^ wt %), a significant improvement of 15% for the average efficiency value and increased stability is demonstrated in non encapsulated devices under high-stress conditions. Impedance spectroscopy reveals that graphene induced an enhancement in recombination resistance, *R*_rec_. These results are explained in terms of the modifications induced by graphene in the analyzed structural, morphological, optical, and electronic properties of the G-MAPbI_3_ thin films. This work demonstrates a simple route to obtain graphene/metal halide composites that can pave the way to the development of low-cost solar cells.

## 2 Results and Discussion

### 2.1 Structural and Morphological Characterization

The used graphene platelets correspond to few-layer graphene with defects mainly related to the domain size according to its Raman spectrum (Figure S1, Experimental Section in the Supporting Information). The narrow widths of G and 2D peaks reveal the superior quality of this graphene^36^ compared to that of GO and rGO materials. For fundamental characterization, MAPbI_3_ thin films on glass substrates were obtained with different graphene contents in the precursor solution (see the Experimental Section of the Supporting Information). The samples are named as 0G (reference sample without graphene), 2.5G, 5G, 10G, 50G, and 100G, corresponding to 0, 2.5, 5, 10, 50, and 100 mg/L of G in the precursor solutions of MHPs, equivalent to 0, 0.6, 1.2, 2.4, 12, and 24 × 10^–3^ wt % graphene content in the films. The thickness of the films is given in Table S1.

**Figure 1:**
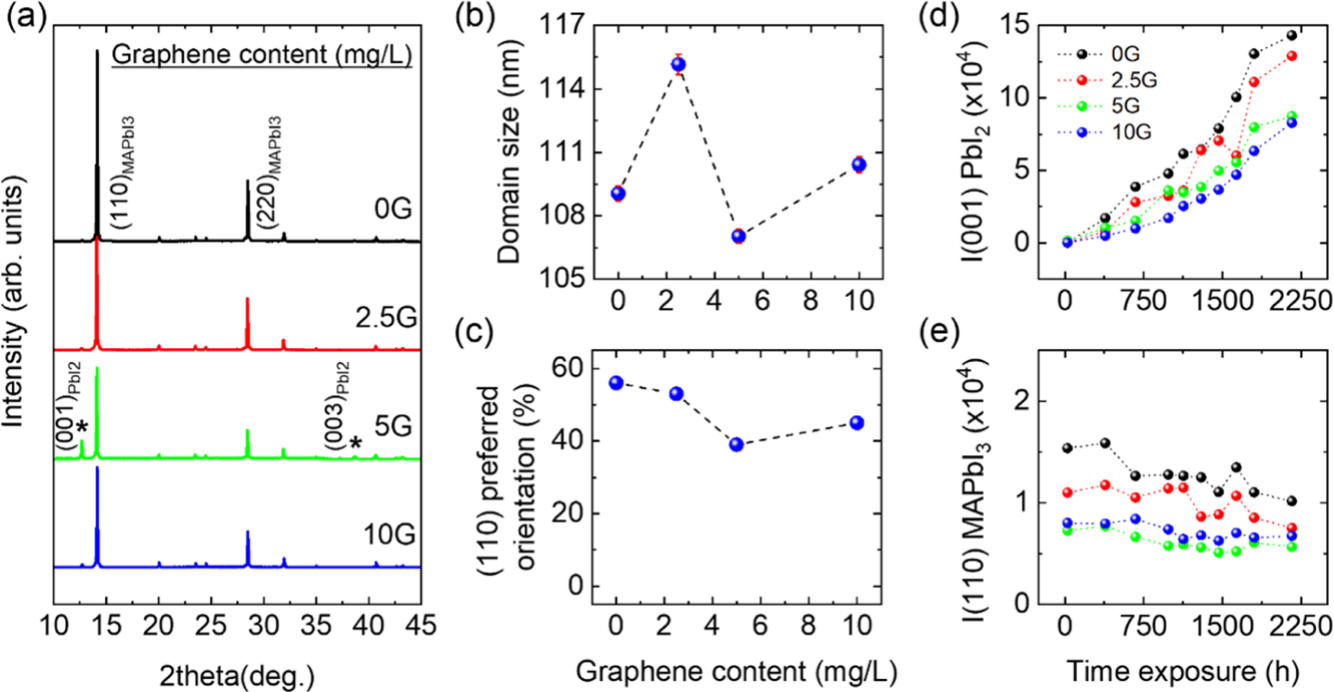
(a) XRPD profiles (Cu Kα1/α2) of the 0G–10G MAPbI_3_ films at 300 K. The asterisks indicate the impurity of PbI_2_. (b) Variation of the average crystal domain size of the MAPbI_3_ films with the graphene content. (c) Preferred orientation-graphene concentration relationship of the MAPbI_3_ films. Time evolution of (d) (001) reflection intensity of the PbI_2_ degradation product and (e) (110) reflection intensity of MAPbI_3_.

Figure 1a shows the X-ray powder diffraction (XRPD) profiles for the reference film and for those with increasing amounts of G-platelets. The samples are polycrystalline films of tetragonal MAPbI_3_, indexed to an *I*4*cm* tetragonal cell,^11,37^ with an important fraction of (110)-oriented grains. All samples contain a minor amount of PbI_2_ impurity. The tetragonal-perovskite lattice parameters were refined by a profile matching approach (Figure S2) obtaining values of 8.865(1) Å and 12.661(4) Å for *a* and *c*, respectively. These values do not change when graphene is added, showing that, as expected, the perovskite composition is not modified.

The average crystal domain size in the films has been carefully obtained using the Williamson-Hall (WH) methodology (See Figure S3 in the Supporting Information). This method allows determining the crystal domain size and strain with great precision since the whole diffraction pattern is used instead of a single peak. The domain size for the smallest amount of graphene, 2.5G film, shows an increase of approximately 8 nm. Such an increase (∼10%) is reliable considering the very small error (red whickers in Figure 1b) provided by the standard deviations shown in Figure S3. Besides, derived from the WH analysis, it can be stated that the films are free of microstrain, in view of the small slopes of the straight lines used to fit the data in Figure S3. On the other hand, Figure 1c shows the dependence of the (110) preferred orientation on the graphene content in the perovskite films. It is clearly observed that the reference film and the one with the lowest amount of G-platelets, 2.5G, present a high degree of alignment (55–60%) compared to a powder sample; however, a further increase in the amount of graphene results in a decrease in the fraction of aligned crystals (40–45%) and therefore an increase in the polycrystalline fraction.

The stability of the films under ambient conditions (25 °C, 40–60% RH) over time has been followed by XRPD (Figure S4). The degradation of the films can be followed by the evolution of the intensities of (001) PbI_2_ and (110) MAPbI_3_ reflections with time (Figure 1d,e). The evolution of the PbI_2_ (001) reflection with time may lead to conclude that the PbI_2_ content increases to large proportions and that there is a large degradation of the perovskite films. However, it has to be highlighted that to quantitatively analyze the diffraction patterns, the almost complete (001) preferred orientation of PbI_2_, that provides a greatly enhanced intensity of the (00*l*) maxima, has to be considered. In fact, the degree of degradation of the perovskite phase is small even after 2000 hours, as confirmed by the intensity of the (110) MAPbI_3_ reflection which decreases only a little with time (Figure 1e). The role of graphene in the perovskite stabilization is evidenced by the time evolution of both peaks (Figure 1d,e). After up to 2000 hours, the degradation of the films is hampered by the introduction of graphene and more efficiently as graphene concentration increases.

### 2.2 Optical Absorption and Photoluminescence

**Figure 2:**
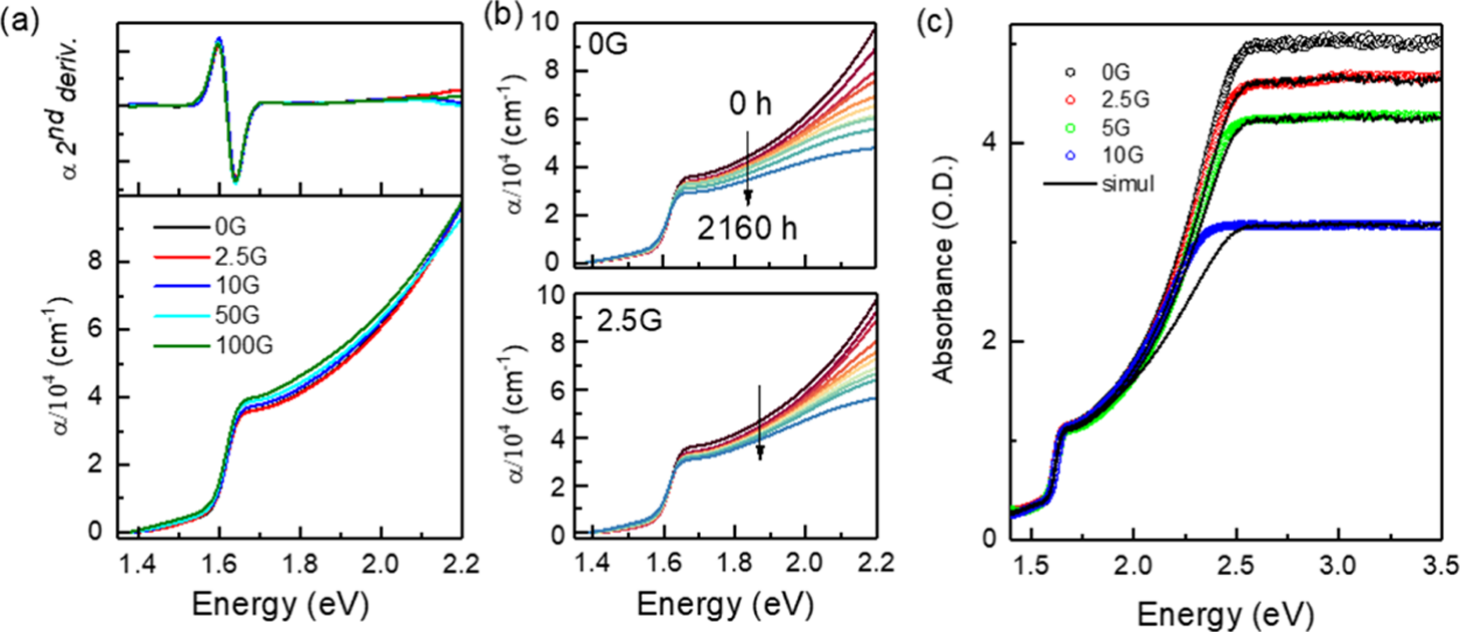
(a) Optical absorption, α, of the thin films (lower panel) and second derivative (upper panel). (b) Time evolution of 0G and 2.5G samples for up to 2160 hours. (c) Raw absorbance data of the 0G–10G films and simulations (black lines, see the text below) of 2.5G, 5G, and 10G considering effective hole fractions per unit area in the films of 10^–60^, 10^–28^, and 10^–6.5^, respectively.

The measured optical absorption spectra of the thin films (Figure 2a) are independent of G doping for the low graphene concentrations, up to 10G, but a slight increase is detected for a higher graphene content (50G and 100G). The optical band gaps were obtained from the second derivatives of the optical spectra (upper panel of Figure 2a)^37,38^ that show a local minima at 775 nm (1.60 ± 0.05 eV) for all films (the full width at half-maximum of the second derivative is assumed as the error) that correspond to the direct semiconductor-type transitions at the R point in the pseudo-cubic Brillouin zone.^3,39^ The absorption spectra have been recorded along ∼2200 hours to explore the evolution of the undoped and G-doped MAPbI_3_ films under ambient conditions; Figure 2b shows the time evolution of 0G and 2.5G samples (see Figure S5a,b for the evolution of absorption spectra for 5G and 10G). With time, the MAPbI_3_ absorption edge amplitudes barely decrease, indicating that the degradation is moderate in all cases but is slightly more pronounced for the 0G film compared to the G-doped films. These results are consistent with the trends of the (110) perovskite diffraction peak upon G incorporation (Figure 1e). Optical absorption results thus confirm that G-platelet addition in the perovskite precursor solution benefits the ambient stability of the thin film, delaying its degradation.

The plateaus typically observed in the high-energy range of absorbance spectra for MAPbI_3_ films with thickness in the range of the samples (>500 nm) studied here are due to the combination of two factors: (1) the weakness of the signal that reaches the detector above the gap (the signal through the samples is 10^OD^ lower than that of the reference channel) and (2) the presence of “pinholes” in the film (or regions with smaller thickness). In Figure 2c, the raw spectra for the 0G–10G films in the spectral region of glass (the substrate) transparency are shown. The plateau, above 2.5 eV, of the 0G film is observed at OD ∼ 5 that is around the detection limit of the detector. However, in the other samples, the plateau appears at lower OD values, thus revealing the presence of pinholes. The optical transmission of a thin film with pinholes is equivalent to a continuous film with, in parallel, a hole of a certain area. In fact, what most probably occurs is the presence of microscopic regions in the films with significantly lower thicknesses than the average, rather than pinholes across the 500 nm. The simplest approximation to take this into account is considering one effective hole in the film. The continuous lines in the figure correspond to the spectra resulting from combining the 0G spectrum in parallel with a hole-effective fraction *A*_h_ = fraction of the film without MAPbI_3_. The fits for 2.5G and 5G spectra using *A*_h_ = 10^–60^ and 10^–28^, respectively, are excellent. These are extremely small hole-effective fractions in the films but unambiguously demonstrate that graphene induces some increasing porosity. For the 10G film, the equivalent hole fraction is significantly higher, *A*_h_ = 10^–6.5^, and the approximation is not so good since it cannot adequately fit the region from 2 to 2.5 eV; however, it shows that the porosity is drastically increased. Therefore, while graphene delays degradation, it also modifies the morphology of the grains and their packing within the film increasing progressively its porosity. Scanning electron microscopy (SEM) images (Figure S5c,d) confirm that porosity increases with the addition of graphene platelets. Moreover, since graphene is an impermeable membrane to gases,^40^ it may be partly sealing the surface of the grains and we hypothesize that could hamper ionic diffusion.

**Figure 3:**
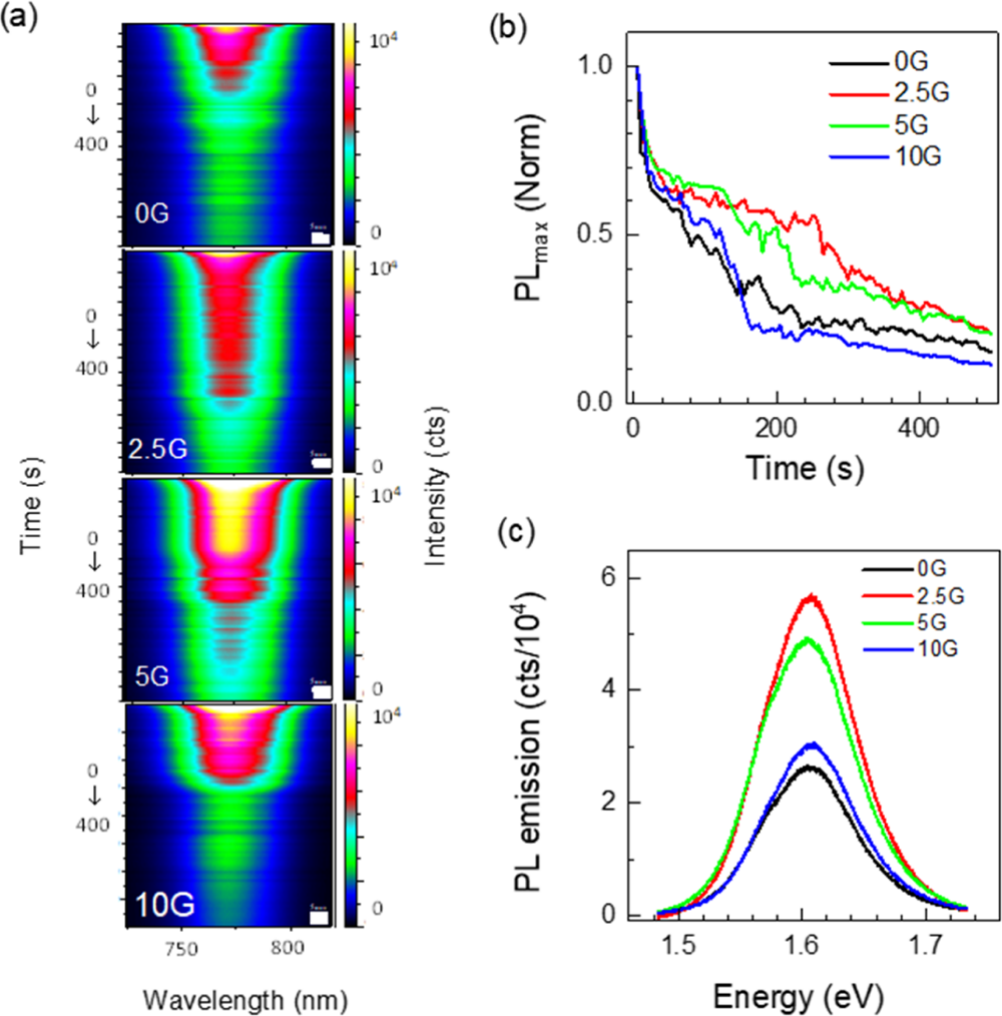
(a) PL spectra evolution with time up to 400 s for the undoped MAPbI_3_, 0G, and G-doped MAPbI_3_ as 2.5G, 5G, and 10G thin films under irradiation with λ_exc_ = 488 nm and *P*_inc_ = 18 W/cm^2^. (b) Normalized time evolution of the PL maximum intensity during 500 s and (c) PL emission spectra at 250 s of irradiation.

Regarding photoluminescence (PL), the addition of G does not induce significant changes in the emission of the samples; all present the characteristic band at an identical energy: 1.60 eV (775 nm) (Figure S6). However, graphene affects the evolution of PL intensity with irradiation time (Figure 3a). The time evolution of PL intensity during 500 s under continuous 488 nm wavelength laser irradiation (18 W/cm^2^) for samples 0G–10G is shown in Figure 3b. The PL emission in MHPs has been extensively studied and reported to exhibit a complex evolution with illumination, time, environmental conditions, and the nature of the layer on which the perovskite grows.^11,41^ The presence of defects, particularly halide vacancies, photoinduced defects and, in general, trap states and their evolution with the factors described above, plays an important role in the nonradiative processes influencing the recombination dynamics.^42,43^ In an earlier work,^11^ we identified four main stages under ambient conditions that MAPbI_3_ evolves under laser irradiation by measuring the PL and the concomitant thin-film Raman spectra. A decrease in the PL intensity under ambient conditions corresponded to the appearance of Raman modes associated with PbO_*x*_, that therefore, is indicative of the beginning of degradation that eventually results in the total fading of the PL emission.

Low and intermediate graphene concentrations provoke less and slower quenching of the PL emission with time (Figure 3b), retaining more than 50% of the initial emission intensity at 250 s under high-power-density laser irradiation under ambient conditions, while 0G thin-film emission retains only 25% of the initial value. Figure 3c shows the PL emission spectra at 250 s. In the case of the 10G film, the evolution with time is better than 0G at short times, but after ∼180 s, it is similar to the film without graphene (0G). The better performance of 2.5G and 5G films under irradiation is consistent with the proposed partial graphene sealing of the grains. However, the limited efficiency of the films with a higher graphene content indicates a trade-off between a better sealing of the grains with the incorporation of graphene and an increasing porosity of the films that facilitates the access to ambient oxygen and water.

### 2.3 Resistivity and Activation Energy

We have studied the effect of G-platelet incorporation on the dark dc resistivity, ρ, of MAPbI_3_ thin films with temperature across the tetragonal to cubic structural transition. All measurements exhibit Ohmic behavior (Figure S7) and a value at 303 K of 2.2 ± 0.2% MΩ·cm for the 0G sample, within published values for MAPbI_3_,: 6.6,^44^ 51,^45^ or 38 MΩ·cm^46^ for polycrystalline samples and 13 MΩ·cm^46^ in single crystals.

**Figure 4:**
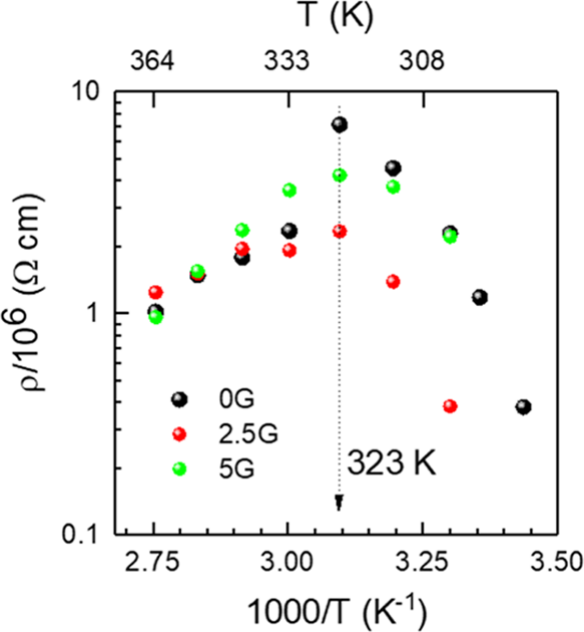
Arrhenius plots for resistivity of the 0G, 2.5G, and 5G MAPbI_3_ thin films from 298 to 373 K (25–100 °C). The dotted arrow indicates the temperature turning point at 323 K as a guide to the eye.

The variation of resistivity in the 25–100 °C (298–373 K) temperature range is shown in Figure 4 (Arrhenius plot). All thin films exhibit similar behaviors. However, the 2.5G MAPbI_3_ thin film shows slightly lower resistivity values for the tetragonal phase, *T* < 333 K. The temperature of the observed turning points in resistivity (at ∼323 K) is very close to the structural, tetragonal (*I*4*mcm*) to cubic (*Pm*3̅*m*), transition temperature, *T*_c_ = 328 K,^47^ pointing out to a correlation between the resistivity behavior and the crystalline phase transition. The impact of structural transitions on optical,^48−50^ thermal,^51^ dielectric,^52^ and photovoltaic^53^ properties of MHPs has been investigated and the electronic structures for both crystalline structures of MAPbI_3_ were calculated.^54^ In general, experiments show relatively minor effects at the cubic–tetragonal transition; however, previous works^55−58^ reported a significant conductivity increase at the tetragonal to orthorhombic low-temperature transition, around 160 K, related to the disorder-order character of the transition, due to MA cation ordering.^47^ The tetragonal to cubic transition has also been described as a disorder-order transition in several perovskites;^59,60^ so, a similar effect on resistivity cannot be ruled out. In fact, this uncommon behavior for a metal-semiconducting crossover with temperature was observed by Głowienka et al.^61^ for dark conductivity, σ, in MAPbI_3–*x*_Cl_*x*_ thin films between 280 and 340 K and by Sveinbjörnsson et al.^62^ between 293 and 353 K under illumination in MAPbI_3_ crystals.

The activation energy (*E*_a_) can be obtained for the cubic phase from the expression for thermally activated conduction, , where ρ_0_ is a pre-exponential factor and *k*_B_ and *T* are the Boltzmann constant and the temperature of the sample, respectively. *E*_a_ values of 0.28, 0.24, and 0.46 eV for undoped 0G and doped 2.5G and 5G MAPbI_3_ thin films are obtained, respectively. In the tetragonal phase, the resistivity increases with temperature (from 298 to 320 K), which is the opposite trend to that of a semiconductor. The structural transition may have an impact on the electronic conduction in a temperature range so close around *T*_c_, but ionic conduction has to be considered since it has an important role in MHPs. The degree of ionic contribution to the charge transport in MHPs is controversial. In all-inorganic halide perovskites, it is commonly accepted that halide-ion conduction via the vacancy diffusion mechanism is the dominant conduction process, with reported activation values in the range of 0.2–0.9 eV.^63−65^ For MAPbI_3_, *E*_a_ calculated from first principles is reported to be 0.58 eV for iodine vacancies,^66^ quite larger than the *E*_a_ values obtained here in the cubic phase, so electronic conduction seems to be dominating dc transport. Very low graphene concentration (2.5G) lowers the hopping activation energy *E*_a_, while larger amounts hinder this hopping transport mechanism (*E*_a_ increases). The increase of the crystalline domain size is induced by the addition of low graphene concentration in the 2.5G sample, while maintaining the fraction of (110) oriented crystals (Figure 2) reduces the density of grain boundaries facilitating intergrain hopping. However, for the 5G sample, grain size and preferential orientation degree are depleted, and the porosity slightly increases compared to undoped MAPbI_3_ films, hampering hopping.

### 2.4 Devices: Solar Cells

#### 2.4.1 Photovoltaic Characteristics and Stability

To evaluate the influence of the G-platelet content (0G–100G) in the active layer of solar cells on the photovoltaic performance, different concentrations of G-platelets in the MAPbI_3_ have been tested in inverted cells with the structure: ITO/PEDOT:PSS/MAPbI_3_:G/PCBM/BCP/Al (details are given in the Supporting Information). The photovoltaic parameters, that is, short-circuit photocurrent (*J*_SC_), open-circuit voltage (*V*_OC_), fill factor (FF), and PCE, were measured under ambient conditions and AM 1.5G solar simulator with a light intensity of 100 mW/cm^2^. The statistical distribution of these parameters and representative *J*–*V* characteristics are shown in Figure S8, and the data of the average and champion devices in reverse and forward scans are collected in Table S2. Undoubtedly, the lower amount of G (2.5G) in the MAPbI_3_ active layer leads to an improvement of ∼6.6% in the final PCE of 2.5G-based solar cells compared to that without graphene (0G). The graphene content above 10G in the active layer induces important photocurrent losses, probably associated to the observed poor crystalline quality of the samples^67−69^ and the increasing porosity of the films with the G content. Interestingly, the *V*_OC_ value does not decrease indicating that energy level matching for charge extraction does not suffer significant alterations.^18^ To summarize, a low graphene content in the active layer (2.5G: 0.6 × 10^-3^ wt % G/PVK) induces, as previously indicated, larger crystalline domains, high preferential orientation, and an increased conductivity, all contributing to an improvement in the final efficiency of the device.

**Figure 5:**
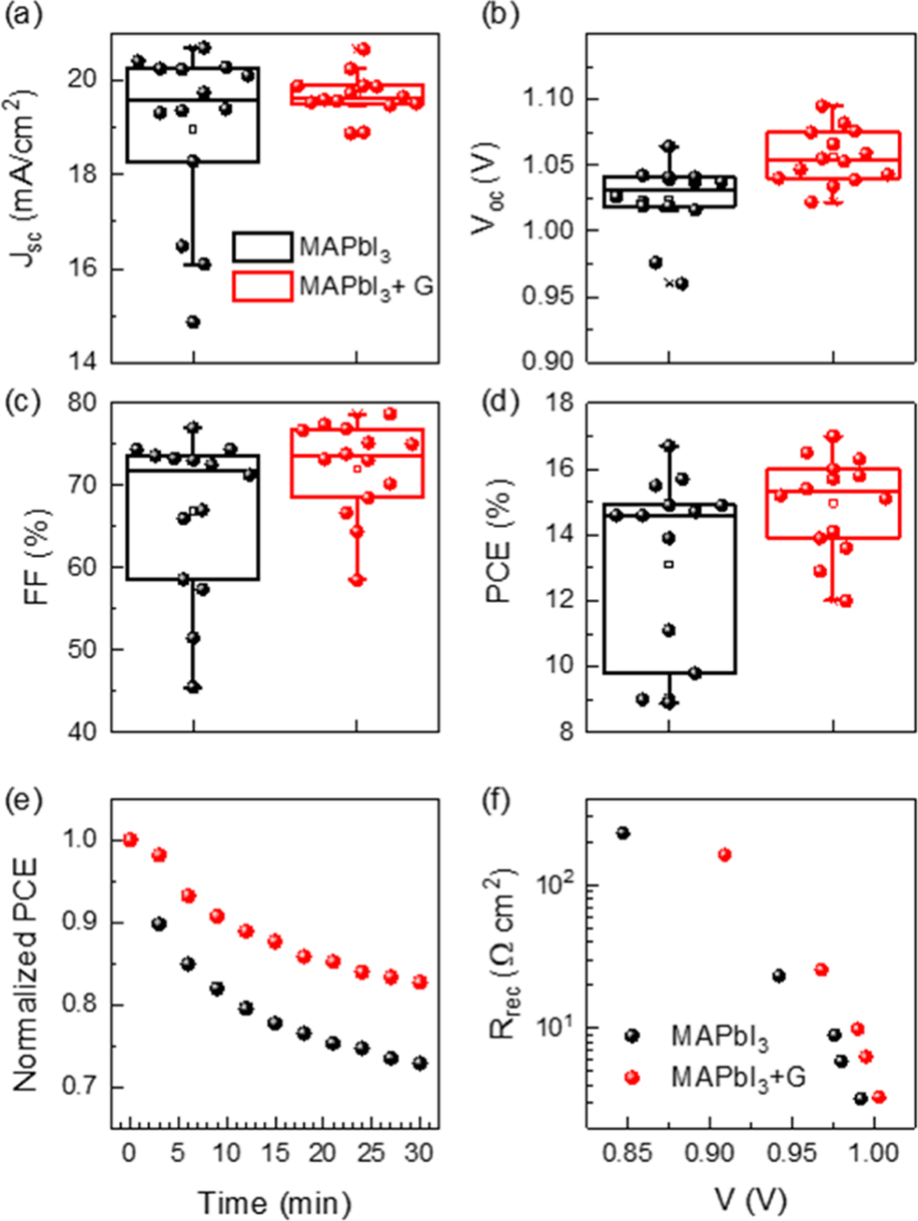
(a–d) Statistical results, average value (open square), and standard deviation of the resulting photovoltaic parameters for ITO/SnO_2_/perovskite/spiro-OMeTAD/Au devices with 0G (no graphene) and with graphene, 2.5G active layers. (e) Normalized PCE evolution of the non encapsulated device under continuous AM1.5G illumination under ambient conditions (*T* = 25 °C, RH ∼ 40–60%). (f) Recombination resistances, *R*_rec_, from impedance spectroscopy measurements.

Based on these results, we have optimized the devices with the 2.5G graphene content in the structure: ITO/SnO_2_/MAPbI_3_:G/spiro-OMeTAD/Au, (see the Supporting Information for experimental details). Comparison of solar cell parameters, *J*_SC_, *V*_OC_, FF, and PCE, of photovoltaic devices using active MHP layers with and without graphene is shown in Figure 5a–d, respectively, and summarized in Table S3. Graphene addition increases the reproducibility and averaged values of all the photovoltaic parameters, especially *V*_OC_. There is a relative increase in the average PCE of 15% for the 2.5G sample over the reference cell without graphene. *J*–*V* curves of champion devices without graphene, 0G, and 2.5G are plotted in Figure S9a. There is a good agreement between the photocurrent measured in the *J*–*V* and calculated from incident photon current efficiency measurements (Figure S9b). We observed again that the average crystal domain is increased with graphene addition, as shown in Figure S10.

Interestingly, graphene addition also has a positive effect on the stability of the PSCs as expected from the fundamental characterization commented above. Devices were tested under high stress conditions using non encapsulated samples and constant AM1.5G lighting for 30 min under ambient conditions with a relative humidity of 40–60%. The presence of graphene has a positive influence on the stability of the solar cell. Figure 5e shows that the solar cell based on a 2.5G active layer retains 83% of its original efficiency, while without graphene, the efficiency is reduced to 73%. *V*_OC_ time evolution for both samples is almost identical, see Figure S11, with a small decrease during the first minutes and a stabilization afterward. However, *J*_SC_ and FF decrease faster for the samples without graphene, see Figure S11.

Impedance spectroscopy experiments were performed on regular 0G and 2.5G PSCs, and measurements were fitted using the equivalent circuit of Figure S12a, according to the model reported in a previous work,^70^ considering the transport resistance in the perovskite layer negligible in comparison with the recombination resistance, *R*_rec_, as it is expected in high-performance PSCs. Two arcs are observed in the Nyquist plot as commonly reported in most of the PSCs, as shown in Figure S12b.^70^Figure 5f shows that *R*_rec_ increases when an optimized amount of graphene is added, 0.6 × 10^–3^ wt %, pointing to a decrease of the nonradiative recombination in the 2.5G PSC, in good agreement with the observed increase in *V*_OC_.

## Conclusions

We tracked the influence of the small graphene platelet content (0–24 × 10^–3^ wt %) on the structural and optoelectronic properties of MHP thin films under ambient conditions and found a double effect that, properly balanced, can improve the device performance and stability of the final solar cell devices. On the one hand, a graphene content of 0.6 × 10^-3^ wt % G/PVK induces the growth of larger crystalline domains maintaining a high preferential orientation, which increases the stability of the MAPbI_3_ films with time under ambient conditions and the endurance to illumination. On the other hand, the porosity of the films that increases with graphene concentration reduces conductivity and could allow the faster diffusion of oxygen and moisture deep into the layer. An appropriated balance tuning graphene content to 0.6 × 10^–3^ wt % G/PVK (2.5G) in the precursor solution produces an increase of all photovoltaic parameters and consequently of the final photoconversion efficiency and delays the degradation, increasing grain size, reducing nonradiative recombination probably by partly sealing the surface of the grains without significantly modifying morphology of the grains and their packing within the film enhancing progressively its porosity. These results open the door to future studies seeking a balance between the processing and efficiency of this composite material through cost-effective perovskite metal halide design strategies aimed at overcoming the stability barrier.

## Supporting Information Available

Experimental details, materials and methods, profile matching fits of XRPD and WH plots, absorbance time evolution, SEM images, photovoltaic performance-graphene content dependence information, EQE and time evolution of photovoltaic performance of solar cells, and equivalent circuit used to fit the impedance data and impedance spectroscopy spectra for 0G and 2.5G-based solar cells at 0.01 mW/cm^2^ of light intensity (PDF)
