# Supplementary material for: Effect of Pristine Graphene on Methylammonium Lead Iodide Films and Implications on Solar Cell Performance
Source: ACS Appl Energy Mater. 2021 Nov 17;4(12):13943–51. doi: 10.1021/acsaem.1c02738 (PMC8715421; doi:10.1021/acsaem.1c02738)
Supplement: Supplementary file 1 — ae1c02738_si_001.pdf [file ae1c02738_si_001.pdf]

Supporting Information

Effect of Pristine Graphene on Methylammonium Lead Iodide Films and Implications on Solar Cell Performance

C. Redondo-Obispo[1], P. Serafini[2], E. Climent-Pascual[3], T.S. Ripolles[4], I. Mora-Seró[2*], A. de Andrés [1*] and C. Coya[4*]

[1] Instituto de Ciencia de Materiales de Madrid, Consejo Superior de Investigaciones Científicas, C/ Sor Juana Inés de la Cruz 3, 28049 Madrid, Spain.

[2] Institute of Advanced Materials (INAM), Universitat Jaume I, 12006 Castelló, Spain.

[3] Escuela Técnica Superior de Ingenieros Industriales, Universidad Politécnica de Madrid, C/ José Gutiérrez Abascal 2, 28006 Madrid, Spain.

[4] Escuela Técnica Superior de Ingeniería de Telecomunicación, Universidad Rey Juan Carlos, C/Tulipán s/n, 28933 Madrid, Spain.

*Corresponding author.

Email: Carmen.coya@urjc.es, ada@icmm.csic.es, sero@uji.es

Experimental Section

Materials and synthesis: All materials were reagent grade and were used as received. Pristine exfoliated graphene flakes suspension in THF (0.1 mg/mL) from GRAnPH Nanotech.[1]

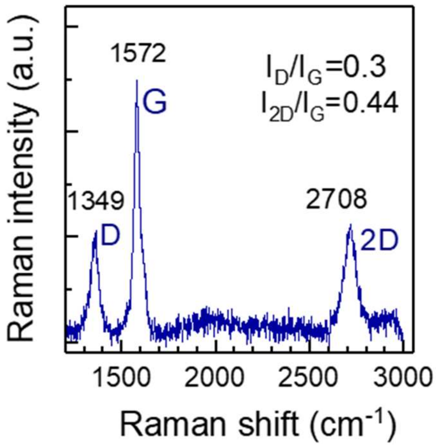

Figure S1. Measured Raman spectra of graphene platelets from GRAnPH Nanotech dispersed in THF.

a) **Inverted structure devices (glass/ITO/PEDOT:PSS/$MAPbI_3$:G/PCBM/BCP/Al)**.

Materials. PEDOT:PSS Clevious P VP AI 4083 aqueous solution from Heraeus. $CH_3NH_3PbI_3$, $MAPbI_3$, synthetized by a stoichiometric mixture of iodine precursors (0.7 M), methylammonium iodide, MAI from Dyesol and lead iodide, PbI_2 from Alfa Aesar (99.999 % ultra dry (metal basis). The anhydrous solvents used were dimethyl sulfoxide (DMSO, >99.8 %, Alfa Aesar) and dimethylformamide (DMF, 99.8 %, Aldrich), DMF:DMSO, 4:1 in v/v. for perovskite solution and toluene (99.8 %, Alfa Aesar) as anti-solvent. The electron transport materials were a fullerene derivative, [6,6]-Phenyl C61 butyric acid methyl ester, PCBM (>99 %, Ossila), solved with anhydrous chlorobenzene (99.8 %, Aldrich), and the electron transport and hole blocking material bathocuproine (BCP, >99.5 %, Ossila), dissolved in anhydrous 2-propanol (>99.5%, Alfa Aesar).

Device fabrication. Pre-pattern ITO on glass substrates (2.5 cm × 3 cm, 15 Ohm/sq, from Lumtech Tech.) were cleaned by ultrasonic bath with acetone, and 2-propanol for 15 min, sequentially, then blow-dried with N_2 gun and ending with an ultraviolet-ozone treatment for 12 min. After this surface treatment, all substrates were transferred to glove box to carry out the fabrication of the solar cells under an inert atmosphere of N_2. PEDOT:PSS HTL, filtered by a 0.45 μm hydrophobic Polyvinylidene fluoride (PVDF) filter, was maintained at 60 °C with the substrate before being spin coated at 4000 rpm for 45 s. Then, the layer was heated for 30 min at 130 °C in a hot plate. The final thickness is 70±2 nm. The precursor solution of $MAPbI_3$ (0.7 M) was prepared by dissolving PbI_2 in DMF:DMSO (4:1, v/v), followed by the addition of MAI to

the PbI_2 solution. The final perovskite solution was filtered by 0.22 μm hydrophobic polytetrafluoroethylene (PTFE) syringe filter. For graphene modified perovskite layers, specified amounts of G dispersions (Table S1) are added to the filtered perovskite solution, and each solution was sonicated 45 min prior to deposition. Solution and substrates were heated at 80 °C before the deposition process. The perovskite film synthesis was carried out by two steps spin coating method. Specifically, $MAPbI_3$ active layers were deposited at 1000 rpm for 10 s, and then, 5000 rpm for 30 s. At 10 s of the second step, toluene was added as anti-solvent in three times the perovskite used volume. The films were kept at room temperature (RT) for 1 h, and then annealed at 100 °C for 10 min in a hot plate. Afterwards, films and 40 mg/mL of PCBM solution were maintained at 60 °C before being spin coated at 2000 rpm for 40 s, followed by a heat treatment at 60 °C for 10 min, resulting in 95±5 nm thickness. At RT, 2 mg/mL of BCP solution was deposited by spin coating at 5000 rpm for 40 s, <10 nm thickness, without subsequent heat treatment. Finally, for the metal electrode, Al was thermally evaporated through a shadow mask at $3 \cdot 10^{-6}$ Torr. Each device area is 25 mm^2, as defined by the overlap between the Al top electrode and the patterned ITO.

Table S1. Sample names of the thin films, graphene concentration in the perovskite precursor solution (in mg/L), amount of graphene in relation to the total amount of perovskite in the precursor solution (weight relation, wt%) and the thickness of the thin films.

**Sample Name**	**[G] in the PVK solution (mg/L)**	**G/PVK wt% ($x10^{-3}$)**	**Thickness (nm)**
0G	0	0	563±5
2.5G	2.5	0.6	551±5
5G	5	1.2	563±5
10G	10	2.4	550±5
50G	50	12	518±5
100G	100	24	534±5

b) **Regular structure devices (glass/ITO/SnO_2/$MAPbI_3$:G/HTL/Au)**.

Materials. All materials were reagent grade and were used as received. Lead iodide (PbI_2, > 98%, from TCI), methylammonium iodide (MAI, 98%, from Greatcellsolar), ethanol (96%) and acetone (99.25%) from PanReac, hydrochloric acid (HCl 37%), dimethyl sulfoxide (DMSO anhydrous 99.9%), clorobenzene (CB anhydrous 99.8%), acetonitrile (MeCN anhydrous 99.8%), 4-tert-butylpiridine (TBP 96%), zinc powder (99.995%) and lithium bis(trifluoromethylsulfonyl)imide (Li-TFSI 99.95%) from Sigma Aldrich, while 2,20,7,70-tetrakis [N,N-di(4-methoxyphenyl)amino]-9,90-spirobifluorene (spiro-OMeTAD 99% from

Feiming Chemical Limited), and SnO_2 colloid precursor from Alfa Aesar (15% in H_2O colloidal dispersion). Indium tin oxide (ITO) coated glass substrates (Pilkington TEC15, ~ 15 Ω sq^{-1}).

Device fabrication. Substrates (indium tin oxide) were etched by using zinc powder and pour over it drops of HCl 2 M. Then glass was cleaned with deionized water, acetone and ethanol in an ultrasonic cleaner for 15 min for each solvent. After being dried by air flow, the substrates were put in an ultraviolet–ozone for 15 min to remove organic residues. Once cleaned, the electron transport layer ETL (SnO_2) was deposited from dissolution of SnO_2 3% from colloid precursor Alfa Aesar. Then, it was spin-coated onto the ITO substrates with a speed of 3000 rpm for 30 s, and heated at 150° C for 30 min. Once the ETL was prepared substrates were submitted to 20 min of UV-Ozone previously to perovskite deposition. Solution was composed of PbI_2, MAI (1.4 M for each) in 4:1 DMF:DMSO (v/v) in one case while other solution with same perovskite and appropriate graphene content for 2.5G sample (5.22 µg/mL G corresponding to $0.6 \cdot 10^{-3}$ wt% G/PVK), was made. Latter solution was previously heated at 70 °C and sonicated for 45 min prior to deposition. Then, a quantity of 80 µL perovskite was deposited over SnO_2 ETL by one-step spin coating at 4000 rpm for 20 s e dripping chlorobenzene antisolvent over perovskite at 8 sec from the beginning of spin coating. As soon as the spin coating was finished, sample was moved to a hotplate and annealed for 10 min at a temperature 100 °C. After perovskite, 80 µL of hole transporting layer made with spiro-OMeTAD in chlorobenzene (85.5 mg/mL) doped with 28.8 µL of TBP and 17.8 µL of a stock solution of 520 mg mL^{-1} of Li-TFSI in acetonitrile was spin-coated at 4000 rpm for 20 s onto the top annealed perovskite layers as hole transport material (HTM). Finally, Au electrode with a thickness of 80 nm was deposited by thermal evaporation.

Film and Device characterization.

Structural characterization. The x-ray powder diffraction (XRPD) profiles of the $MAPbI_3$ thin films were registered by Bruker D8 Advance diffractometer using Cu Kα radiation over a 2θ range between 8° and 65° with a step size of 0.02°. The average crystal domain size in the films, was determined using the extracted FWHM values from the XRPD profiles and the Williamson-Hall (WH) methodology. The layer thickness and roughness were measured using an Alpha Step 200 profilometry (KLA-Tencor Instruments) and analyzed with the Apex 3D software (KLA-Tencor). **SEM** topographic images were acquired with a FEI Verios 460 microscope (13 pA and 2 kV for current and voltage respectively).

Optical Characterization. UV-VIS absorption of the thin films was characterized using a UV-VIS-NIR spectrophotometer (Varian, Cary 5000) in the wavelength range of 300-900 nm. To estimate the optical transition energies, we use the second derivatives of optical spectra, since the minima correspond to absorption maxima and therefore to electronic transitions. Steady

state photoluminescence emission (PL) of the films and micro-Raman spectra of graphene flakes were carried out using the 488 nm excitation wavelength of an Ar+ laser in backscattering geometry (P_{inc}= 18·W/cm^2) with an Olympus microscope with a x20 objective, a "super-notch-plus" Filter from Kaiser and a Jobin-Yvon HR-460 monochromator coupled to a Peltier cooled Synapse CCD.

Electrical measurements. All measurements were carried out in ambient conditions (40-50 % of relative humidity (RH)). Dark resistivity-temperature dependence was obtained from current-voltage characteristics with two-wire configuration due to the high resistance of the samples and a homemade Faradaic box. The dependences with increasing temperature have been measured in the 298-373 K range, with 10 K step size. The samples (thin films on glass substrate) were located on a hot plate with a K–type thermocouple right beside the sample to a process controller Electemp-TFT (Selecta). Current-voltage curves were measured at each temperature after 3 min stabilization, using a probe station and a Keithley 2450 Sourcemeter.

Photovoltaic parameters for non-encapsulated p-i-n devices were obtained by current-voltage measurements performed under AM 1.5G (100 mW/cm^2) conditions with a Wavelabs Sinus-70 AAA LED solar simulator with a Keithley 2450 sourcemeter. Shadow mask to define an area of 9 mm^2 are used. Scan rate of 90 mV/s was carried out without preconditioning. The spectrum of the solar simulator is monitored with reference intensity sensor in the test plane in combination with fast feedback loop for automatic intensity correction and temperature control for the LEDs. **Photovoltaic parameters** for non-encapsulated n-i-p devices were obtained using Abet sun 2000 solar simulator which gives an AM 1.5G (100 mW/cm^2) light illumination conditions coupled with a Keithley 2612 sourcemeter to measure current-voltage. Each measure was taken with Tracer software and done at ambient conditions (T=25 ºC, RH~40-60 %) using a scan rate of 10 mV/s and a shadow mask of 0.121 cm^2.

EQE measurements were performed using a Xenon lamp with a monochromator Oriel Cornestone 130 which was used to measure along the wavelength of the spectrum. Prior measurement, calibration was done using a reference photodiode of silicon and each measurement was obtained using TRACQ BASIC software. Finally, EQE scans were taken from 300 nm to 810 nm in steps of 10 nm.

The impedance spectroscopy (IS) was measured using a Potentiostat Autolab PGSTAT30 using different filters to change light intensity up to 1 sun. For each voltage point (V_{OC}) IS was measured with an AC 10mVvoltage perturbation from 1 MHz to 100 mHz. Nova software was used to generate data and Z-View software for modelling the equivalent circuit model used to fit the spectra, respectively.

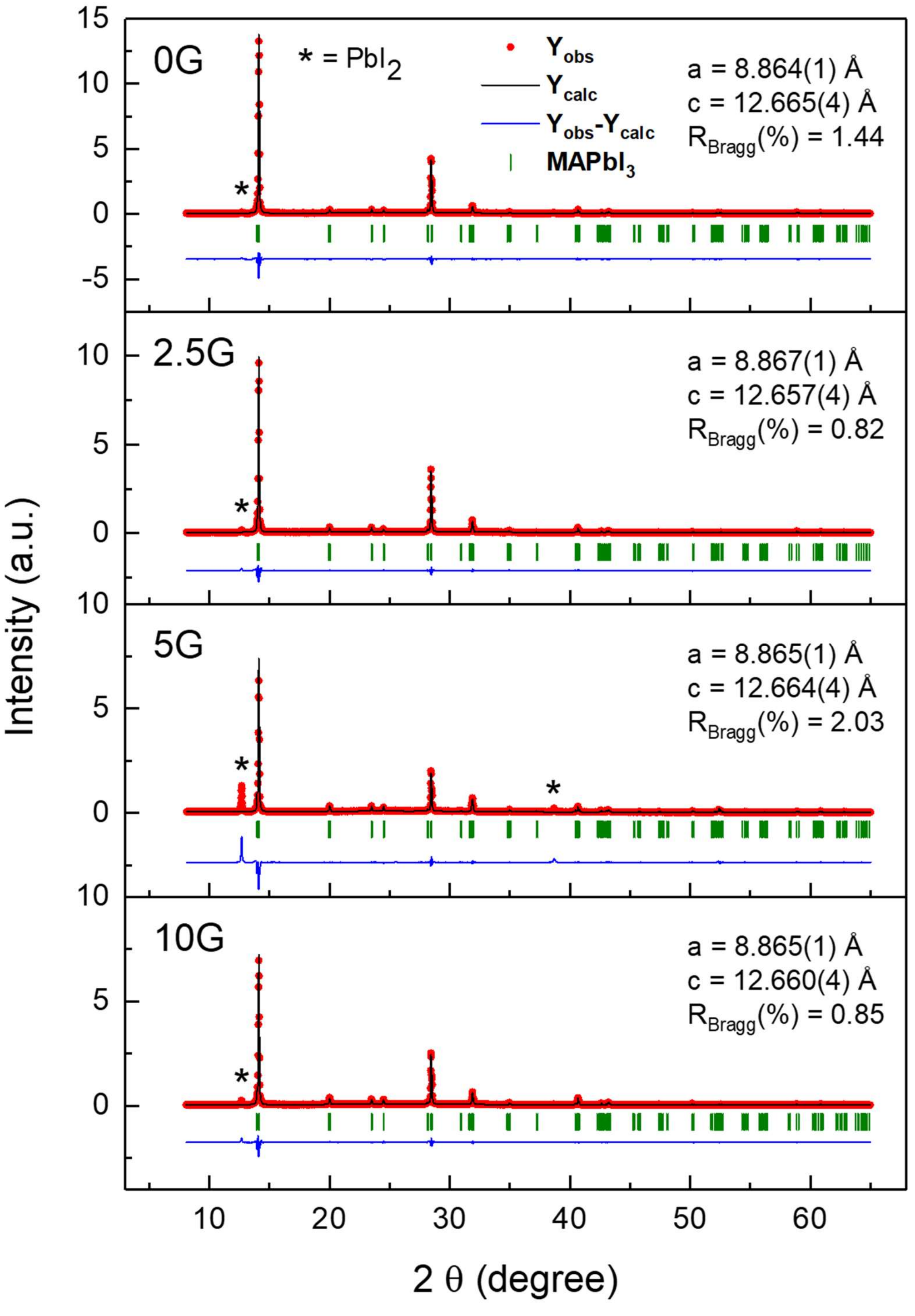

Figure S2. Profile matching fits (solid black line) of XRPD (Cu Kα1/α2) data (red circles) of the films at RT. Green tick marks indicate the position of allowed $MAPbI_3$ (*I*4*cm*). The Bragg R-factor (%) for $MAPbI_3$ is also included. Profile matching made using FullProf software.

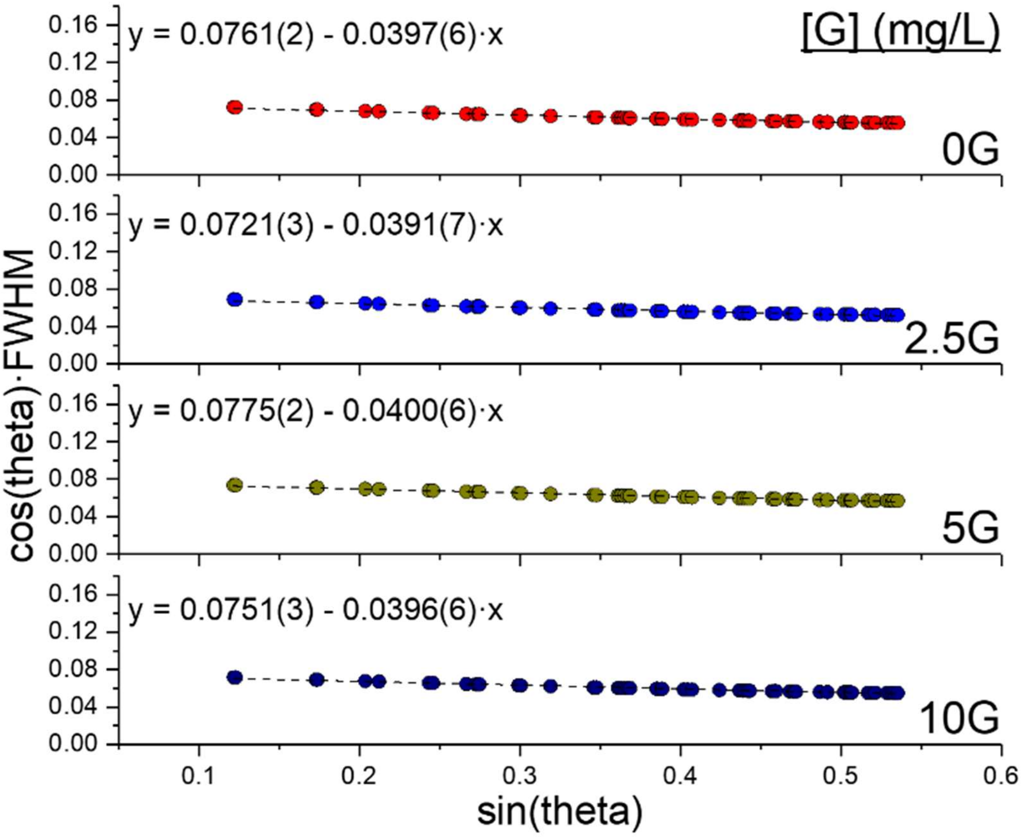

Figure S3. Williamson-Hall plots for the films (0G – 10G).

The domain size and microstrains evolution as a function of graphene platelets content are analyzed by the Williamson- Hall method using the expression,

$$FWHM_{hkl}(2\theta) = \frac{K\lambda}{D\,cos(\theta)} + 4\varepsilon\frac{sin(\theta)}{cos(\theta)}$$

where D represents the domain size in Å, K represents the shape factor (0.9), λ represents the X-ray wavelength 1.5406 Å, FWHM represents the full width at half maximum related to diffraction maximum, θ represents the Bragg's diffraction angle and ε represents the microstrains (%).

If the diffraction maxima width is only caused by crystallite size, $FWHM_{hkl}\times cos(\theta)$ is independent of sin(θ), while when the microstrains have a certain effect on the peak width, the slope of Williamson-Hall plot is not zero. Thus, the microstrains are estimated by the slope of the linear fit of $FWHM_{hkl}\times cos(\theta)$ vs sin(θ) data and the average domain size using the intercept.

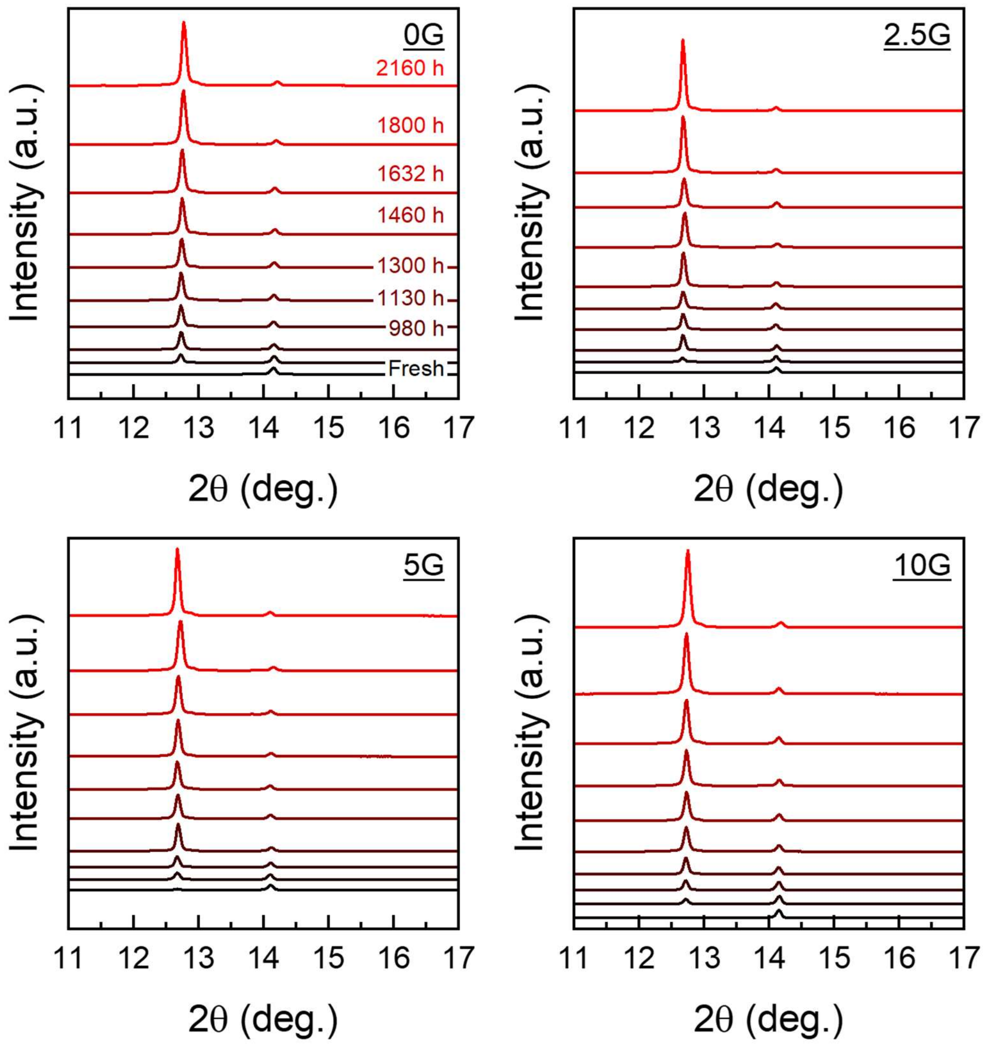

Figure S4. XRPD time evolution for $MAPbI_3$ thin films 0G, 2.5G, 5G and 10G.

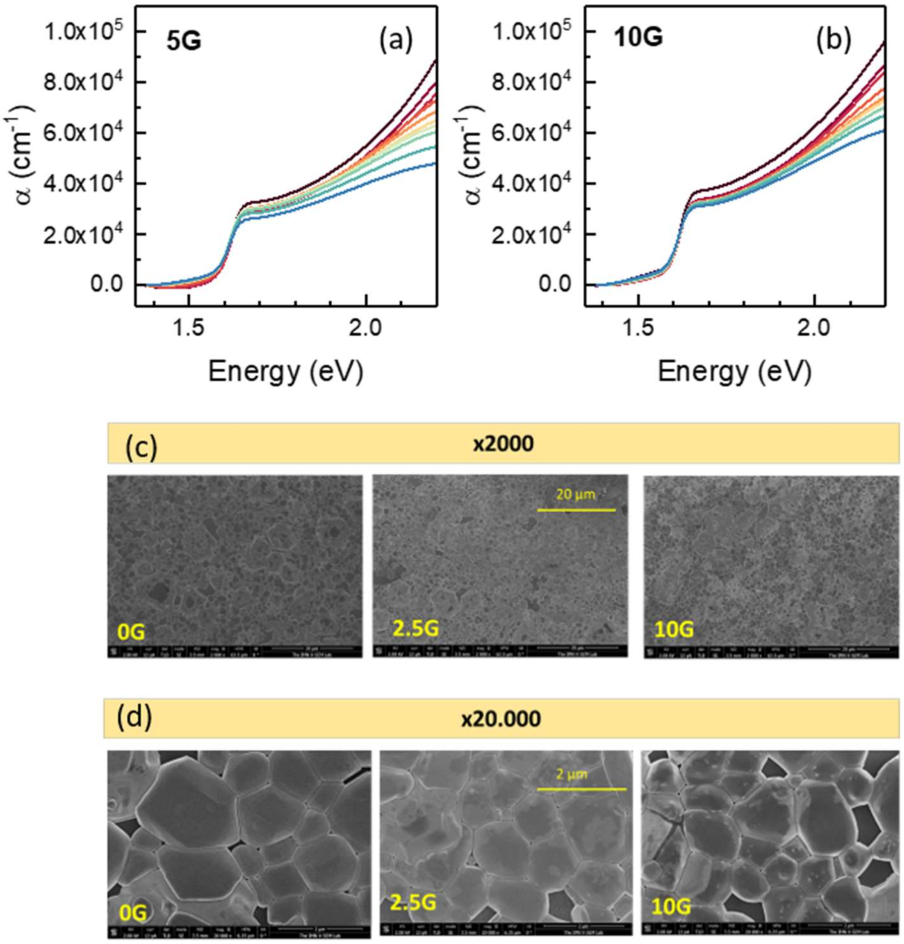

Figure S5. Evolution of absorption spectra for (a) 5G and (b)10G thin films. The blue arrow indicates the time evolution. (c) SEM images at two magnifications, (c) x2000 and (d) x20.000, of 0G, 2.5G and 10G thin films.

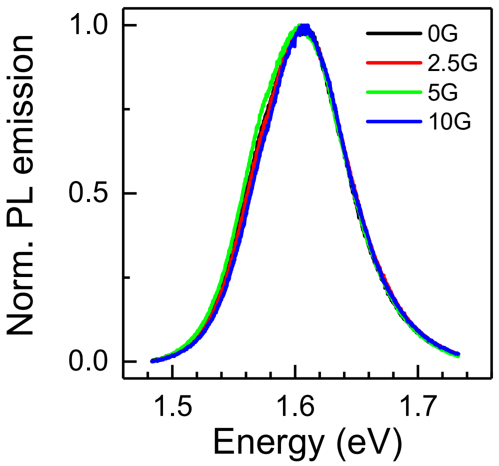

Figure S6. Normalized PL emission of 0G, 2.5G, 5G and 10G.

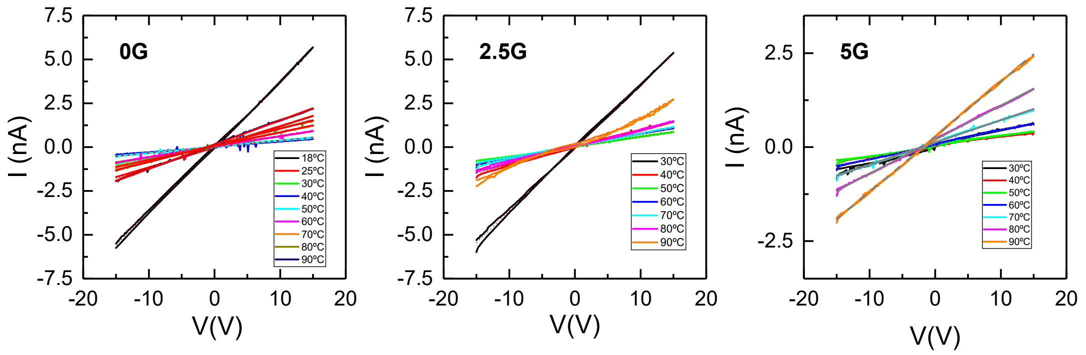

Figure S7. Current-voltage characteristics variation with temperature for 0G, 2.5G, and 5G $MAPbI_3$ films.

The I-V curves are measured through a known volume, defined by the length (L) and the separation (W) between evaporated Al contacts and thin film thickness (z). ρ is obtained from current-voltage characteristic at each temperature. W/L aspect ratio (2.5) and Z (thickness) << W ensure the perpendicular flow of current through an area A=L·Z and a uniform electric field. The measured resistance, R from I-V curves, allow us to calculate the resistivity from: $R = \rho \frac{w}{A}$.

Graphene content variation into glass/ITO/PEDOT:PSS/MAPbI$_3$:G/PCBM/BCP/Al solar cells.

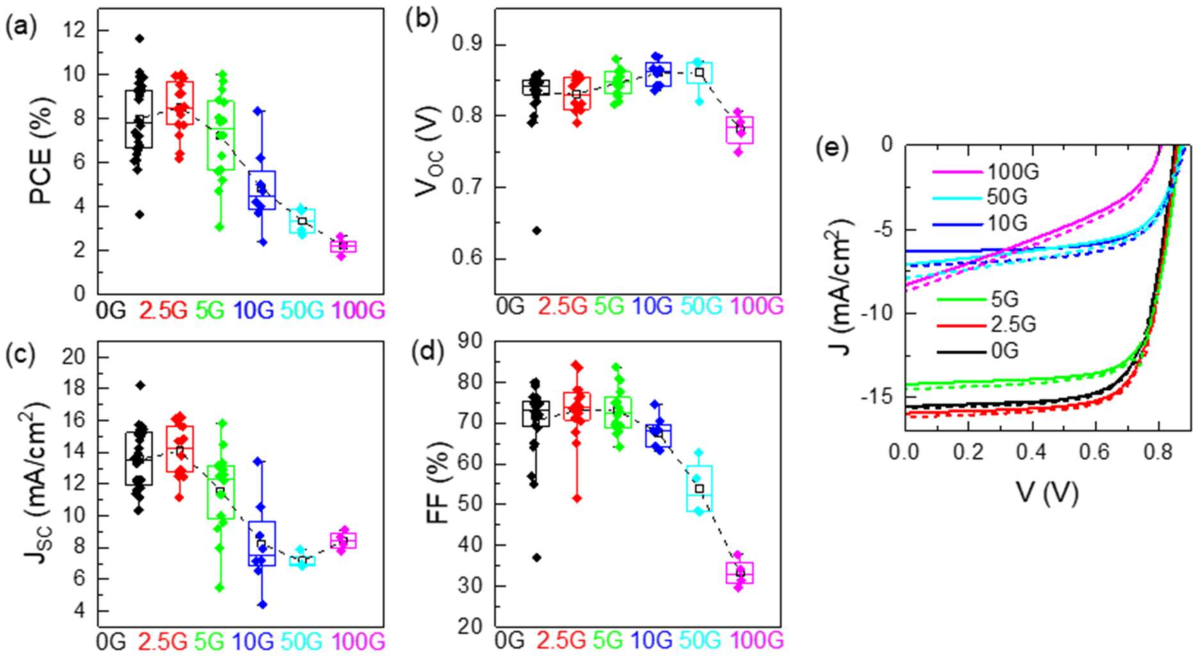

Figure S8. (a)-(d) Statistical distributions of photovoltaics parameters under AM1.5G in reverse scans of glass/ITO/PEDOT:PSS/MAPbI$_3$:G/PCBM/BCP/Al solar cells, using 0G, 2.5G, 5G, 10G, 50G and 100G. as active layers. Dashed lines are a guide to the eye. (e) Representative current density−voltage curves of the PSCs tested devices (solid and dashed lines for forward and reverse scans respectively).

Table S2. Photovoltaic performance of $MAPbI_3$ based PSCs without and with increasing graphene flakes content in reverse/forward scan.

Samples $MAPbI_3$		PCE (%)	V_{OC} (V)	J_{SC} (mA/cm^2)	FF (%)
0G	Average	7.99±1.76/7.88±1.62	0.83±0.04/0.83±0.05	13.56±1.97/13.26±1.97	70.60±8.95/71.44±9.18
	Champion	11.63/11.43	0.84/0.84	18.23/18.01	76.33/75.85
2.5G	Average	8.52±1.22/8.19±1.13	0.83±0.02/0.83±0.02	14.08±1.62/13.92±1.51	73.03±7.65/71.10±7.82
	Champion	10.01/9.85	0.86/0.86	16.15/15.93	72.47/72.22
5G	Average	7.22±1.97/6.99±1.92	0.85±0.02/0.85±0.02	11.55±2.60/11.04±2.70	72.98±5.50/74.39±4.76
	Champion	10.02/9.82	0.86/0.86	15.81/15.55	73.72/73.41
10G	Average	4.83±1.79/4.47±1.75	0.86±0.02/0.86±0.02	8.24±2.74/7.43±2.87	67.56±3.89/70.10±2.96
	Champion	8.36/7.76	0.84/0.84	13.41/12.98	74.66/71.62
50G	Average	3.33±0.60/3.03±0.66	0.86±0.03/0.85±0.04	7.15±0.50/6.35±0.51	53.89±7.05/55.75±8.10
	Champion	3.90/3.68	0.88/0.87	7.91/7.11	56.40/59.52
100G	Average	2.20±0.37/2.00±0.34	0.78±0.02/0.77±0.03	8.44±0.59/7.99±0.55	33.25±3.45/32.20±3.38
	Champion	2.64/2.45	0.81/0.80	8.68/8.31	37.73/36.66

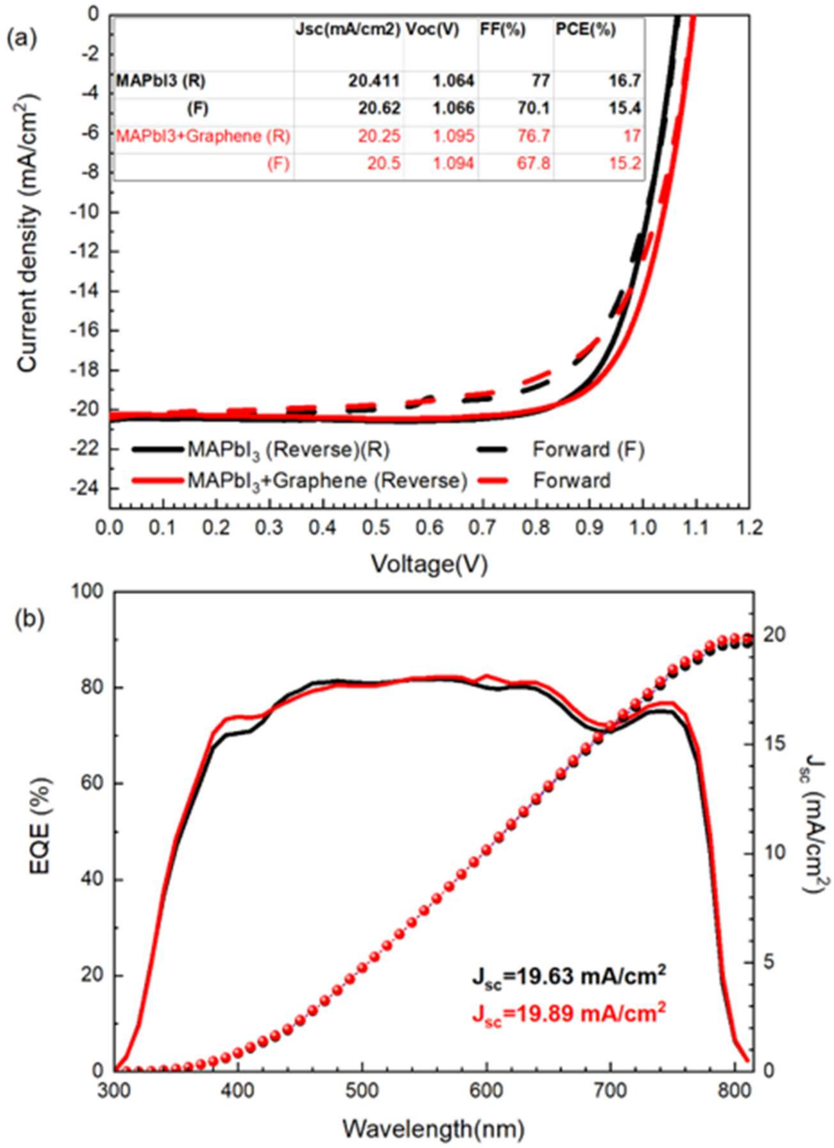

Figure S9. (a) JV curve of champion n-i-p devices measured in forward (F) and reverse (R) direction with its respective parameters. (b) EQE measurement of champion devices.

Table S3. Photovoltaic performance of n-i-p $MAPbI_3$ based PSCs without G and using 2,5G for average in reverse scan and for champion device in reverse/forward scan.

		J_{SC} (mA/cm^2)	V_{OC} (V)	FF (%)	PCE (%)
**Average**					
$MAPbI_3$		18.06±0.99	1.02±0.01	66.83±5.23	13.09±1.52
$MAPbI_3$+G		19.67±0.25	1.06±0.01	72.02±3.07	14.96±0.78
**Champion device**					
$MAPbI_3$	Reverse	20.41	1.06	77.00	16.67
	Forward	20.62	1.07	70.10	15.40
$MAPbI_3$+G	Reverse	20.25	1.10	76.70	17.03
	Forward	20.50	1.09	67.80	15.21

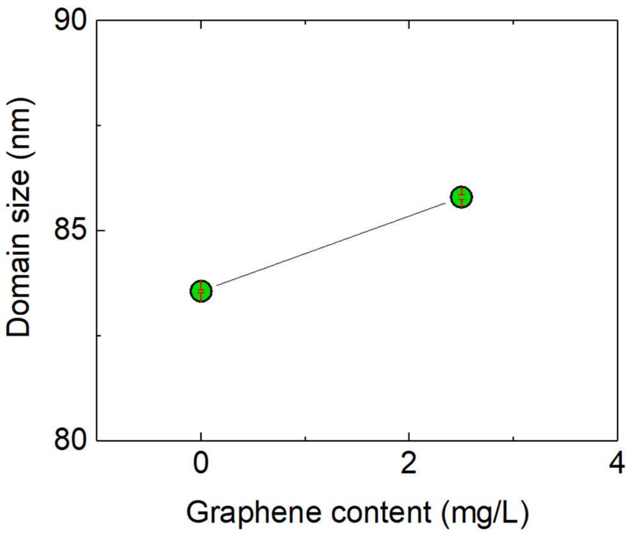

Figure S10. Variation of the average crystal domain size of the synthesised $MAPbI_3$ films for n-i-p devices with graphene content.

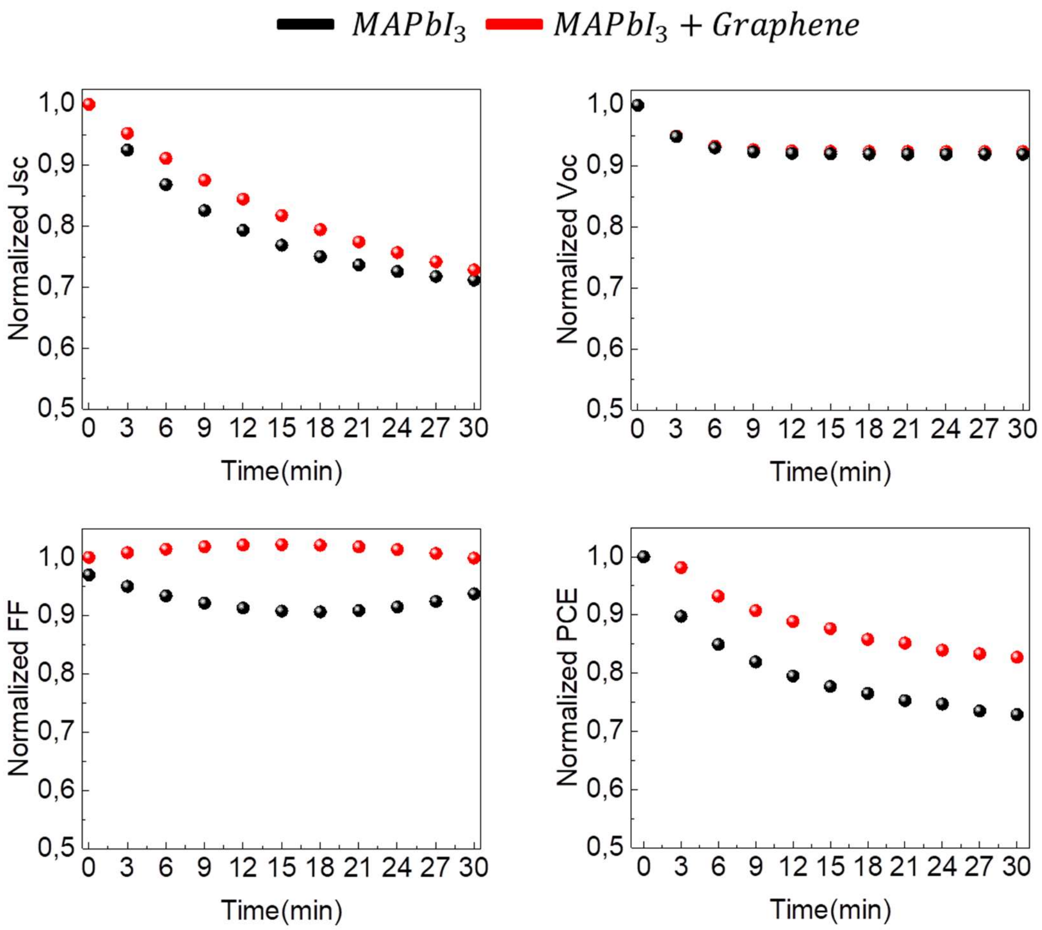

Figure S11. Parameter results of stability measurement over 30 minutes under 1 sun illumination constantly at ambient conditions of 25 °C and 40-60 % RH for non-encapsulated n-i-p solar cells, for no graphene (0G) and with graphene (2.5G) active layers.

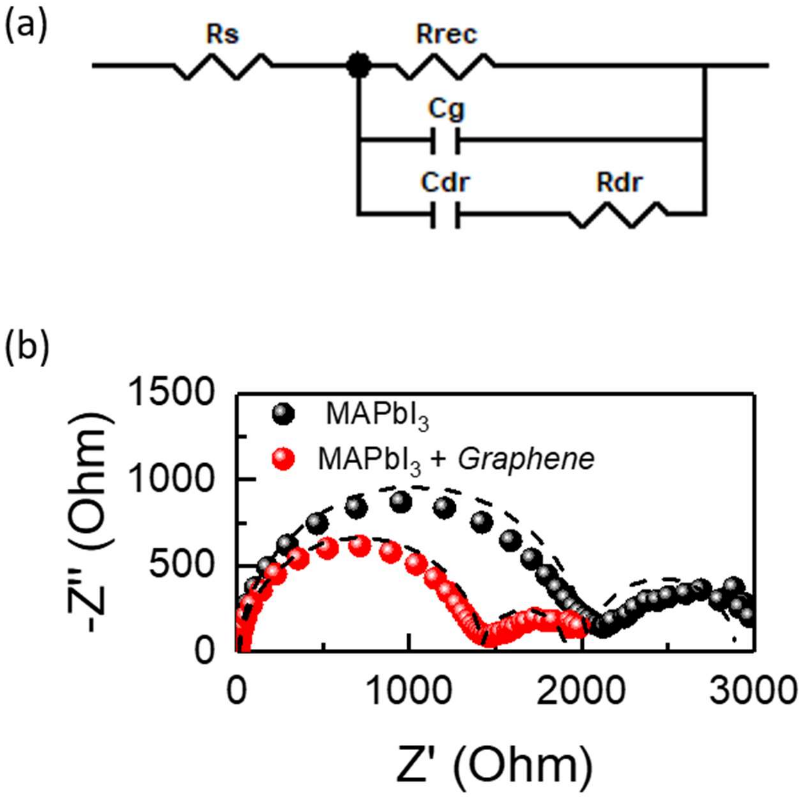

Figure S12. (a) Equivalent circuit used to fit the impedance data.[2] R_s represents the series resistance due to the wires and contacts, R_{rec} is the recombination resistance. We consider that transport resistance is negligible in comparison with R_{rec}. C_g is the geometrical capacitance. The branch containing C_{dr} and R_{dr}, a capacitor and a resistor respectively, is the responsible of the splitting of the pattern in two arcs, where C_{dr} is the low frequency capacitance. Note that ideal capacitors have been used for the fitting in order to avoid constant phase elements with unphysical interpretation. (b) Impedance spectroscopy spectra for 0G and 2.5G-based solar cells at 0.01 mW/cm^2 of light intensity. The dashed line fits the Nyquist plots using the equivalent circuit model represented in (a).

[1] A.E. Del Rio-Castillo, C. Merino, E. Díez-Barra, E. Vázquez, Selective suspension of single layer graphene mechanochemically exfoliated from carbon nanofibres, Nano Res. 2014, 7 (7), 963–972

[2] Yoo, S.-M.; Yoon, S. J.; Anta, J. A.; Lee, H. J.; Boix, P. P.; Mora-Seró, I., An Equivalent Circuit for Perovskite Solar Cell Bridging Sensitized to Thin Film Architectures. Joule 2019, 3 (10), 2535-2549.
